# Multifunctional Roles of *Bacillus* spp. in Sustainable Agriculture: Advances in Biocontrol, Omics, and Ecological Applications

**DOI:** 10.1007/s00248-026-02709-2

**Published:** 2026-02-03

**Authors:** Mateti Gayithri, Srishti Singh, Bhubaneswar Pradhan, Venkataramana Boorla, Sasmita Chand

**Affiliations:** 1 Assam Agricultural University, Jorhat, 785013 Assam India; 2 CMP Degree College, University of Allahabad, Prayagraj, 211002 Uttar Pradesh India; 3 Division of Agricultural Biotechnology, School of Agriculture and Rural Development, Ramakrishna Mission Vivekananda Educational and Research Institute, Narendrapur Campus, Kolkata, 700103 West Bengal India; 4 Manipal School of Architecture and Planning, Manipal Academy of Higher Education, Manipal, 576104 Karnataka India

**Keywords:** *Bacillus*, Biocontrol, PGPR, Sustainable agriculture, Microbial ecology, Omics

## Abstract

The intensifying demand for sustainable agriculture necessitates eco-friendly alternatives to synthetic agrochemicals. *Bacillus* spp., a versatile group of plant-associated rhizobacteria, have emerged as potent biocontrol agents with multifaceted roles in promoting crop health and resilience. This review synthesizes recent advances in the ecological and functional understanding of *Bacillus* spp., emphasizing their mechanisms of action, including antibiosis, siderophore production, biofilm formation, induction of systemic resistance, and secretion of lytic enzymes. The capacity of *Bacillus* to modulate phytohormone levels, improve nutrient acquisition, and mitigate abiotic stresses underpins their role as effective plant growth-promoting rhizobacteria (PGPR). Moreover, their ability to combat a broad spectrum of phytopathogens, fungal, bacterial, viral, nematodal, and insect pests, highlights their biocontrol potential in diverse agroecosystems. Advances in omics technologies have unraveled critical genes and regulatory pathways governing these beneficial traits, enabling targeted genetic engineering for enhanced efficacy and environmental adaptability. Despite their widespread applicability, gaps remain in formulation stability, field performance consistency, and regulatory harmonization. This review underscores the need for integrative research combining microbial ecology, genomics, and precision agriculture to optimize *Bacillus*-based biocontrol strategies for climate-resilient and sustainable crop production.

## Introduction

Agriculture faces an urgent need for sustainable strategies to enhance crop production in the face of growing population demands, pest and disease pressures, and environmental constraints. Plant pathogenic microbes alone are estimated to cause roughly 20–25% yield losses [1], and intensive reliance on chemical fertilizers and pesticides has led to environmental degradation and health concerns. In this context, microbial biocontrol agents have gained prominence as efficient and eco-friendly alternatives to synthetic agrochemicals. Among these, *Bacillus* spp. have risen to the forefront as versatile bioinoculants that can simultaneously protect plants from pathogens and promote growth. Several *Bacillus*-based biopesticides and biofertilizers (notably strains of *Bacillus thuringiensis *(Bt), *Bacillus subtilis*, *Bacillus amyloliquefaciens*, *Bacillus velezensis*, etc.) have been developed and commercially adopted worldwide [2]. The global biocontrol market, valued at about US$4.0 billion in 2020, is projected to exceed $10 billion by 2027, reflecting the increasing reliance on microbial solutions like *Bacillus* in crop management [3].

*Bacillus* species are gram-positive, spore-forming bacteria ubiquitously present in soil and rhizosphere environments. Their ability to form endospores confers extreme tolerance to heat, desiccation, and other stresses, making them resilient inoculants that can survive field conditions and long-term storage [4]. All *Bacillus* spp. are prolific producers of secondary metabolites, including antibiotics, lipopeptides, enzymes, and volatile organic compounds (VOCs), which enable them to antagonize a wide range of plant pathogens and pests [5]. In parallel, these bacteria engage in beneficial interactions with plants as plant growth-promoting rhizobacteria (PGPR), for example, by producing phytohormones (such as indole-3-acetic acid and gibberellins) that stimulate root development, or by fixing nitrogen and solubilizing phosphorus to enhance nutrient availability [6]. *Bacillus* spp. also helps plants tolerate abiotic stresses (drought, salinity, etc.) by modulating stress-responsive pathways and producing osmoprotectant compounds. These multifaceted capabilities have earned *Bacillus* the reputation of biological toolboxes in agriculture, capable of simultaneously improving crop health, yield, and soil quality [2].

Notably, *B. thuringiensis* (Bt) has long been used as a microbial insecticide, revolutionizing pest control in crops through its production of Cry toxins specific to insect larvae. The success of Bt exemplifies the potential of *Bacillus* spp., and recent studies show that other *Bacillus* strains can similarly target fungal and bacterial diseases, even some plant viruses indirectly, thereby contributing to integrated disease management [7]. Furthermore, advancements in high throughput omics technologies have deepened our understanding of how *Bacillus* interacts within the phytobiome. Genomic sequencing and comparative genomics have identified gene clusters responsible for antifungal and growth-promoting compounds. Transcriptomic and proteomic analyses reveal how *Bacillus* gene expression and proteins change in response to pathogen or plant signals, shedding light on biocontrol mechanisms. Metabolomic profiling has led to the discovery of novel antimicrobial metabolites and elicitors of plant defence. These insights are driving the development of genetically improved *Bacillus* strains and more effective formulation technologies [8].

In this paper, we provide a structured examination of the current knowledge and recent advances (primarily from 2020 to 2025) regarding the roles of *Bacillus* spp. in sustainable agriculture. First, discuss *Bacillus* as biocontrol agents, detailing their modes of action against diverse plant pathogens and pests, and propose a reorganization of the concept of disease management under the broader umbrella of biocontrol. Then, explore their plant growth-promoting activities and contributions to soil health. Next, we delve into molecular and omics-driven advancements, including genetic engineering efforts to enhance *Bacillus* traits, and how these align with ecological applications. Finally, we address the environmental impacts, safety, and practical considerations of deploying *Bacillus* in the field, and conclude with future prospects and research needs. By synthesizing these aspects, we aim to underscore the potential of *Bacillus* spp. as cornerstone microbes in the transition toward more sustainable, resilient, and productive agricultural systems.

## *Bacillus* as Biocontrol Agents

### Mechanisms of *Bacillus* Biocontrol

*Bacillus* spp. employ a suite of mechanisms to suppress plant pathogens and pests, making them effective biocontrol agents in various cropping systems. Key modes of action include:

#### Antibiosis through Antimicrobial Metabolites

**Fig. 1:**
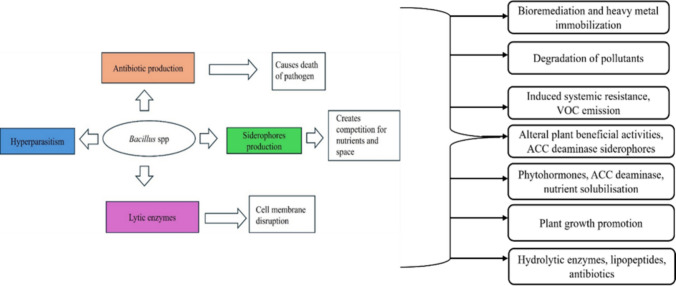
**S**chematic representation of the multifaceted plant beneficial mechanisms of *Bacillus* spp.

**Fig. 2:**
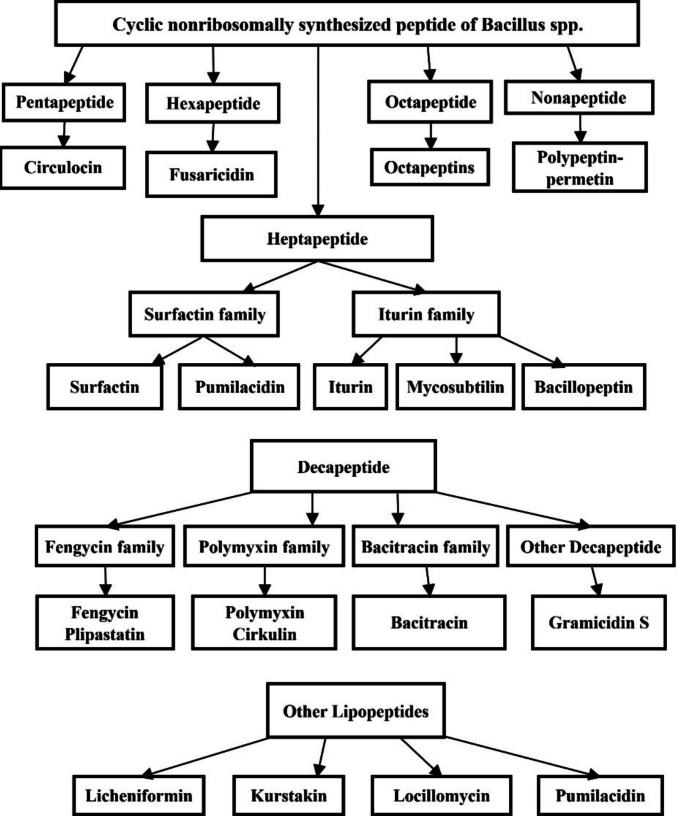
**S**chematic representation of the diversity of antibiotics produced by *Bacillus* spp.

One hallmark of *Bacillus* biocontrol is the production of potent antimicrobial compounds that directly inhibit pathogens. Many strains synthesize antibiotics and lipopeptides such as *iturins*, *fengycins*, *surfactins*, *bacillomycins*, and others, which can disrupt the cell membranes or vital processes of fungi and bacteria [9]. For example, *iturin* and *fengycin* lipopeptides insert into fungal cell membranes, causing leakage and cell death, as evidenced by significantly reduced fungal growth in *Bacillus*-treated plants [10]. Various mechanisms of *Bacillus* spp. are depicted in Fig. 1. Besides, the diversity of antibiotics produced by *Bacillus* spp. is depicted in Fig. 2. Separately, *Bacillus amyloliquefaciens* NCPSJ7 showed effective antifungal activity. CR analysis confirmed the presence of key lipopeptide biosynthetic genes (srfAA, sfp, fenD, bmyB, ituD, and ituC) [11]. C-MS identified three lipopeptide families: *iturin A*, *fengycin A*, and *surfactin*, with C14-iturin A being the primary antifungal compound, as determined by ESI-HRMS. 14-iturin A inhibited fungal mycelial growth and conidial germination at 30 μg/mL [12]. EM and TEM analyses revealed significant alterations in fungal structure, including damage to the outer cell wall, swollen mitochondria, increased vacuolation, and compromised membrane integrity. PI staining and conductivity tests confirmed increased membrane permeability, likely due to iturin A's interaction with ergosterol. These findings suggest that *B. amyloliquefaciens* NCPSJ7 and its lipopeptides, particularly iturin A, are promising biocontrol agents due to their potent antifungal mechanisms [13].

In addition, *Bacillus* produces extracellular lytic enzymes such as chitinases, β−1,3-glucanases, and proteases that degrade the cell walls of fungal pathogens and the biofilms of bacterial pathogens [14]. The secretion of these enzymes further weakens invading pathogens and complements the chemical antibiosis effect. In addition, these antimicrobial compounds inhibit plant pathogens and other soil microbes, making *Bacillus* a dominant species in the rhizosphere [15].

#### Competition for Nutrients and Niche Exclusion

*Bacillus* can outcompete pathogens in the rhizosphere by rapidly colonizing plant roots and consuming available resources. This competition is a crucial biocontrol mechanism, as it directly affects the survival and proliferation of pathogens in the rhizosphere [16]. Siderophore production by *Bacillus* spp. is a critical mechanism for inhibiting pathogen growth. These high-affinity iron-chelating compounds sequester iron from the environment, making it unavailable to competing pathogens [17]. The diversity of siderophores produced by *Bacillus*, each with varying affinities for iron, enhances its ability to compete for this essential nutrient under diverse environmental conditions [18, 19]. This nutrient competition strategy is a crucial biocontrol mechanism in iron-poor rhizosphere environments. Additionally, by occupying physical space on root surfaces and forming protective biofilms, *Bacillus* establishes a barrier that is difficult for pathogens to penetrate [20]. Biofilm-derived exopolysaccharides can bind to root tissue and further prevent pathogen attachment while immobilizing nutrients away from pathogens. Such competitive exclusion is significant for suppressing soil borne diseases [21].

#### Induced Systemic Resistance (ISR) in Plants

The induction of plant defence responses by *Bacillus* involves the production of signaling molecules that activate defence-related pathways, enhancing the plant's resilience against pathogenic attacks [22]. This induced systemic resistance (ISR) is complex, involving a network of phytohormones and signalling cascades that result in the production of defence enzymes and structural barriers [23]. Beyond directly attacking pathogens, *Bacillus* spp. can activate the plant’s own defense systems. Certain strains produce signal molecules (such as small lipopeptides, volatile organic compounds, and phytohormone analogs) that trigger ISR, a state of heightened defensive capacity in the plant. *Bacillus*-mediated ISR often operates through jasmonic acid and ethylene signaling pathways, leading to the priming of defense genes and accumulation of pathogenesis-related proteins in plant tissues. *Bacillus* siderophores act as elicitors of plant immunity. Recent research demonstrated that purified siderophores from *B. amyloliquefaciens* can markedly boost plant defense enzyme activities (e.g., peroxidase, polyphenol oxidase, chitinase) and upregulate defense-related gene expression, thereby fortifying the plant against subsequent pathogen attack [24, 25]. These findings underscore that siderophores serve a dual purpose: they deprive pathogens of iron and simultaneously act as molecular signals that *prime* the plant’s immune system for enhanced resistance [25]. The net effect of ISR induction by *Bacillus* is a reduction in disease severity across a range of pathogens, even some (like viruses) that the bacteria do not directly interact with. For instance, treated plants often show higher levels of defensive compounds (phytoalexins, phenolics) and cell wall strengthening, which collectively inhibit pathogen colonization and spread [26].

#### Biofilm Formation and Exopolysaccharide Production

*Bacillus* spp. can form biofilms capable of controlling pathogens through multiple mechanisms. For example, *Bacillus* subtilis is widely recognized as an effective biocontrol agent due to its ability to form robust biofilms [27]. The exopolysaccharides (EPS) produced during biofilm formation serve as physical barriers, preventing pathogens from accessing plant roots and chelating nutrients to limit the availability for competing microbes [24]. Additionally, biofilms help *Bacillus* spp. colonize plant roots, enhancing plant growth and resistance to infection by stimulating the plant’s immune system [28]. Particular species, such as *Bacillus* *amyloliquefaciens*, produce EPS that not only promote biofilm development but also enhance antifungal activity [26]. Furthermore, EPS production aids in environmental stress tolerance, such as survival in acidic rhizosphere conditions [29, 30]. Some *Bacillus*-associated lactic acid bacteria also produce EPS with potential applications in food and human health due to their functional and health-promoting properties [31].

### *Bacillus* in Disease Management

#### Biocontrol of Fungal Pathogens

Spore-forming bacteria of the genus *Bacillus* are highly effective in combating fungal pathogens and play a vital role in sustainable agriculture [30]. Table 1 highlights the targeted fungal pathogens associated with various plant diseases. These bacteria produce several antifungal compounds, iturins, fengycins, and surfactins that disrupt the pathogen’s cell membranes, leading to cell lysis and death. This antibiosis mechanism is particularly effective against soil-borne pathogens such as *Fusarium* spp. and *Phytophthora *spp., which are known for causing significant crop losses [32]. For instance, *Bacillus* *subtilis* inoculation in tomato plants led to an 85% reduction in Fusarium wilt incidence [33], owing to its production of secondary metabolites that suppress fungal growth and enhance plant resistance [34]. Similarly, *Bacillus* *amyloliquefaciens* exhibits vigorous antagonistic activity against *Phytophthora infestans*, the pathogen responsible for late blight in potatoes, by producing lipopeptides that disrupt pathogen cell walls and membranes [35]. Beyond soil-borne pathogens, *Bacillus* spp. also control foliar fungal pathogens such as *Botrytis cinerea*, the causal agent of grey mould in many crops [36]. Their ability to colonize the rhizosphere allows them to competitively exclude pathogenic fungi by occupying ecological niches and depleting available nutrients. This competition is further intensified by siderophore production, which sequesters iron, an essential micronutrient for fungal growth, thus inhibiting pathogen proliferation [37]. Additionally, *Bacillus* spp. induces systemic resistance in plants through the activation of hormonal pathways that stimulate the production of phytoalexins and pathogenesis-related proteins, which reinforce cell walls and inhibit fungal invasion [38]. The production of hydrolytic enzymes such as chitinases and glucanases further degrades fungal cell walls, limiting their capacity to infect host tissues. This multifaceted approach not only protects crops but also contributes to soil health by reducing pathogen load [39].

**Table 1:** Targeted fungal pathogens with various plant diseases

*Bacillus* Spp	Bioactive lipopeptides	Targeted Fungal/bacterial pathogens	plant disease	Reference(s)
*Bacillus amyloliquefaciens*	Iturin/Fengycin	*Aspergillus phoenicis*, *Bipolaris sorokiniana*, *Fusarium oxysporum*	Food spoilage, seedling diseases, Fusarium crown and root rot	[40]
*Bacillus amyloliquefaciens*	Iturin/Surfactin	*Alternaria alternata*, *Aspergillus* spp., *Botryosphaeria obtusa*, etc	Pre- and postharvest diseases in fruits, vegetables, and medicinal plants	[41]
*Bacillus amyloliquefaciens*	Iturin/Surfactin	*Rhizoctonia solani*	Damping-off of soybean	[42]
*Bacillus amyloliquefaciens*	Bacillomycin D/Fengycin	*Fusarium oxysporum* f. sp. *spinaciae*	Fusarium wilt on spinach	[43]
*Bacillus amyloliquefaciens*	Surfactin/Fengycin	*Sclerotinia sclerotiorum*	Sclerotinia stem rot disease	[44]
*Bacillus amyloliquefaciens*	Surfactin/Fengycin	*Penicillium italicum*, *Fusarium culmorum*, *Botrytis cinerea*, etc	Apple rot, Fusarium foot rot, rice blast	[45, 46]
*Bacillus amyloliquefaciens*	Iturin A	*Alternaria citri*, *Colletotrichum gloeosporioides*, *Penicillium crustosum*	Postharvest fungal diseases in fruits	[47]
*Bacillus amyloliquefaciens*	Bacillomycin D	*Fusarium oxysporum* f. sp. *cucumerinum*	Fusarium wilt of cucumber	[48]
*Bacillus amyloliquefaciens*	Bacillomycin L/D	*Rhizoctonia solani*	Damping-off of soybean	[49]
*Bacillus amyloliquefaciens*	Surfactin	*Sclerotinia sclerotiorum*, *Rhizoctonia solani*, *Fusarium solani*	Sclerotinia stem rot, Rhizoctonia rot, pepper root rot	[50]
*Bacillus amyloliquefaciens*	Fengycin	*Verticillium dahliae*, *Fusarium oxysporum*, *Phytophthora parasitica*	Verticillium wilt, Fusarium wilt, pepper root rot	[51]
*Bacillus subtilis*	Surfactin	*Pseudomonas syringae*	Arabidopsis root infection	[52]
*Bacillus subtilis*	Iturin	*Xanthomonas campestris pv. cucrbitae*; *Pectobacterium carotovorum*	Bacterial leaf spot, soft rot	[53]
*Bacillus subtilis*	Iturin/Surfactin	*Xanthomonas campestris pv. campestris*, *X. axonopodis pv. itri*	Citrus canker	[49]
*Bacillus amyloliquefaciens*	Surfactin	*Xanthomonas axonopodis pv. glycines*	Root and foliar diseases of soybeans	[52]

#### Biocontrol of Bacterial Pathogens

*Bacillus spp.* are potent antagonists against a wide range of bacterial pathogens, playing a crucial role in reducing disease incidence in crops. The specific bacterial pathogens associated with various plant diseases are listed in Table 2. By producing antibiotics such as bacillomycin, *Bacillus* disrupts the cellular processes of harmful bacteria, effectively inhibiting their growth and reducing their populations in the rhizosphere. This mechanism is crucial for managing diseases caused by pathogens like *Xanthomonas* spp. and *Pseudomonas* spp., which are responsible for significant crop losses [54]. In addition to antibiotic production, *Bacillus* competes with bacterial pathogens for nutrients and ecological niches in the root zone, thereby suppressing pathogen proliferation and infection potential [55]. This competitive advantage is further strengthened by its ability to rapidly colonize root surfaces and form biofilms, which act as physical barriers protecting plant roots [56]. Moreover, *Bacillus spp.* can trigger systemic acquired resistance (SAR) in plants, enhancing the plant’s innate immune system. This is achieved through the production of signaling molecules that activate defence-related genes, preparing the plant to resist bacterial infections better [57]. Consequently, plants treated with *Bacillus* exhibit enhanced resistance to bacterial diseases, contributing to improved plant health and yield [58]. Furthermore, *Bacillus* produces lytic enzymes capable of degrading bacterial biofilms’ protective matrices that pathogens form to withstand environmental stress and plant defence mechanisms [34]. These enzymes may also enhance the efficacy of other biocontrol agents by disrupting pathogen shielding strategies [59].

**Table 2:** Targeted bacterial pathogens with various plant diseases

*Bacillus* Spp	Bioactive lipopeptides	Targeted bacterial pathogens	Plant disease	References
*Bacillus subtilis*	Surfactin	*Pseudomonas syringae*	Arabidopsis root infection	[52]
*Bacillus subtilis*	Iturin	*Xanthomonas campestris pv. cucrbitae, Pectobacterium carotovorum*	Bacterial leaf spot, soft rot on melon leaves	[60]
*Bacillus amyloliquefaciens*	Surfactin	*Xanthomonas axonopodis pv. glycines*	Root and foliar diseases of soybeans	[52]
*Bacillus amyloliquefaciens*	Iturin/Surfactin	*Xanthomonas arboricola pv. juglandis*	Walnut blight	[41]
*Bacillus amyloliquefaciens*	Iturin	*Pseudomonas syringae pv. aptata*	Sugar beet blight	[42]
*Bacillus amyloliquefaciens*	Iturin A2	*Agrobacterium tumefaciens*, *Erwinia carotovora* subsp. *arotovora, Clavibacter michiganensis* subsp. *ichiganensis, Xanthomonas campestris pv. ampestris*	Crown gall, bacterial wilt, and rot diseases	[61]
*Bacillus subtilis/amyloliquefaciens*	Surfactin/Fengycin	*Ralstonia solanacearum*, *Erwinia amylovora*, *Pseudomonas syringae pv. yringae*, *Xanthomonas arboricola pv. ragariae*, *Xanthomonas axonopodis pv. esicatoria*	Bacterial wilt and rot diseases	[62]
*Bacillus amyloliquefaciens*	Fengycin	*Clavibacter michiganensis* subsp. *ichiganensis*	Bacterial wilt and canker of tomato	[63]
*Bacillus subtilis*	Surfactin	*Pectobacterium carotovorum* subsp. *arotovorum*	Soft rot on plant leaves	[63]
*Bacillus subtilis*	Bacillomycin L	*Agrobacterium tumefaciens*	Crown gall disease	[63]
*Bacillus subtilis*	Iturin	*Xanthomonas oryzae pv. oryzae*	Rice blight	[63]
*Bacillus velezensis*	Fengycin/Surfactin	*Erwinia amylovora*	Fire blight of apples and pears	[64]
*Bacillus licheniformis*	Iturin	*Ralstonia solanacearum*	Bacterial wilt in tobacco and potatoes	[65]
*Bacillus cereus*	Surfactin	*Pseudomonas fluorescens*	Bacterial soft rot	[66]

#### Biocontrol of Viral Pathogens

*Bacillus* spp. plays an indirect yet significant role in managing viral pathogens in crops [67]. Although they do not directly target viruses, *Bacillus* enhances the plant’s natural defences by inducing systemic acquired resistance (SAR), which stimulates the production of defence-related enzymes and proteins that inhibit viral replication [68]. This resistance is facilitated through the production of signaling molecules such as salicylic acid and jasmonic acid, which activate plant defence pathways, strengthen cell walls, and promote the synthesis of antiviral compounds. Consequently, plants treated with *Bacillus* exhibit reduced disease severity and incidence [69]. Moreover, *Bacillus* modulates ethylene levels in plants, an important stress hormone often elevated during viral infections. By maintaining hormonal balance, *Bacillus* minimizes the negative impacts of viral attacks and supports plant growth and resilience [70]. Table 3 presents various plant viral pathogens managed through these mechanisms. Overall, the physiological enhancement brought about by *Bacillus* results in healthier plants that are more resilient to viral stress, contributing to lower infection rates and improved yield outcomes [71, 72].

**Table 3:** Targeted viral pathogens with various plant diseases

*Bacillus* spp.	Bioactive lipopeptides	Targeted viral pathogens	Plant disease	Reference(s)
*Bacillus subtilis*	Surfactin	Tobacco mosaic virus (TMV)	Tobacco mosaic disease	[73]
*Bacillus amyloliquefaciens*	Iturin	Cucumber mosaic virus (CMV)	Cucumber mosaic disease	[43]
*Bacillus velezensis*	Bacillomycin D	Rice yellow mottle virus (RYMV)	Rice yellow mottle disease	[74]
*Bacillus cereus*	Surfactin	Potato virus Y (PVY)	Potato virus Y disease	[75]
*Bacillus amyloliquefaciens*	Iturin A	Zucchini yellow mosaic virus (ZYMV)	Zucchini yellow mosaic	[16]
*Bacillus subtilis*	Surfactin/Fengycin	Banana bunchy top virus (BBTV)	Banana bunchy top disease	[76]
*Bacillus subtilis*	Iturin	Papaya ringspot virus (PRSV)	Papaya ringspot	[77]
*Bacillus licheniformis*	Surfactin	Tomato yellow leaf curl virus (TYLCV)	Tomato yellow leaf curl	[78]
*Bacillus megaterium*	Bacillomycin D	Pepper mild mottle virus (PMMoV)	Pepper mild mottle disease	[63]
*Bacillus thuringiensis*	Iturin	Cabbage leaf curl virus (CaLCV)	Cabbage leaf curl	[79]
*Bacillus pumilus*	Surfactin	Rice tungro bacilliform virus (RTBV)	Rice tungro disease	[80]
*Bacillus amyloliquefaciens*	Iturin/Surfactin	Tobacco leaf curl virus (TLCV)	Tobacco leaf curl	[81]
*Bacillus subtilis*	Fengycin	Groundnut rosette virus (GRV)	Groundnut rosette	[82]
*Bacillus polymyxa*	Surfactin	Bean common mosaic virus (BCMV)	Bean mosaic disease	[83]

#### Biocontrol of Nematodes and Insect Pests

In addition to microbial pathogens, *Bacillus* species are employed as biocontrol agents against invertebrate pests, including plant-parasitic nematodes and herbivorous insects [84]. The soil-borne nature of many *Bacillus* spp. makes them especially suitable for targeting root pests such as nematodes, while spore-forming *Bacillus* can also be formulated as foliar sprays for insect control [85]. For plant-parasitic nematodes (e.g., root-knot nematodes *Meloidogyne* spp., cyst nematodes, etc.), *Bacillus* offers protection through multiple mechanisms. Certain *Bacillus* strains produce metabolites with nematicidal properties; for example, iturins and other toxins can be lethal to nematode juveniles by disrupting their cuticle and digestive tract. Research has shown that culture filtrates of *B. amyloliquefaciens* caused significant mortality of *Meloidogyne incognita* larvae in vitro. *Bacillus* can also reduce nematode infestation by competitive exclusion in the rhizosphere by rapidly colonizing root surfaces. *Bacillus* can occupy the niches where nematodes would usually penetrate and consume root exudates that nematodes might use as chemical cues or food [86]. Moreover, *Bacillus*-induced systemic resistance plays a role: treated plants develop enhanced levels of defensive chemicals in roots (such as phenols and lignin), making them less attractive or susceptible to nematode infection [87].

For instance, a study reported that tomato plants inoculated with selected *Bacillus* strains had fewer root galls and egg masses of *Meloidogyne*, along with evidence of heightened root defenses and reduced nematode fecundity. There are also commercial *Bacillus* products (e.g., *Bacillus firmus* strain I-1582, marketed as a bionematicide) that have shown efficacy in field trials, reducing nematode population densities and improving crop yield in infested soils. These successes illustrate that *Bacillus* can be a viable tool for managing nematodes in an eco-friendly manner, decreasing the reliance on synthetic nematicides [88]. Some *Bacillus* spp. produce toxins that can directly harm nematodes. For instance, researchers found that *Bacillus amyloliquefaciens* and *Lysinibacillus sphaericus* have a significant nematicidal effect against *Meloidogyne incognita* [89]*. Bacillus* can outcompete nematodes for resources in the rhizosphere, limiting their growth and reproduction [90]. Certain *Bacillus* strains can trigger a defence response in plants, making them more resistant to nematode infection [81]. Several studies have demonstrated the effectiveness of *Bacillus* against *Meloidogyne* spp. One study found that selected *Bacillus *spp*.* effectively controlled *Meloidogyne* spp*.* on tomato plants [91]. Another study highlighted the potential of *Bacillus firmus* GB-126 (commercialised as BioNem®) in reducing nematode populations and improving cotton yields in field trials [92].

When it comes to insect pest control, *Bacillus thuringiensis* (*Bt*) is the most famous example, known for its production of crystal (Cry) and cytolytic (Cyt) proteins toxic to specific insect orders [93]. Bt formulations have been widely and safely used for decades to control lepidopteran caterpillars, dipteran larvae (e.g., mosquitoes and blackflies), and some coleopteran pests. The Cry toxins, when ingested by insect larvae, bind to gut receptors and create pores in the midgut cells, leading to paralysis and death of the pest [94]. These toxins are particular; for example, *B. thuringiensis kurstaki* targets many caterpillar pests (such as the diamondback moth, cabbage looper, and armyworms) without harming beneficial insects or mammals [95]. Another strain, *B. thuringiensis israelensis* (Bti), is effectively used to kill mosquito and fungus gnat larvae in water by releasing toxins that disrupt their midgut, thus aiding in vector control. The use of *Bt* has dramatically reduced crop damage in many systems and is a cornerstone of organic insect pest management [96]. Aside from *B. thuringiensis*, other *Bacillus* species also contribute to insect control. *Bacillus subtilis* and *B. pumilus* produce metabolites (e.g., proteases, chitinases, surfactin) that have insecticidal or insect-deterrent effects. For instance, a *B. subtilis* strain secreting a serine protease (Sep1) was shown to degrade the cuticular and gut lining proteins of certain insect pests, weakening their defense barriers [97]. Biosurfactants from *B. subtilis* can damage the midgut epithelium of insects, interfering with nutrient absorption and causing high larval mortality. Some *Bacillus* VOCs act as repellents, reducing pest feeding and oviposition on treated plants [98]. In practice, foliar sprays of non-*Bt Bacillus* strains have been observed to reduce populations of sap-sucking insects like aphids and whiteflies, potentially by a combination of repellent VOCs and mild direct toxicity. For example, *B. subtilis* applied to cereal crops significantly lowered aphid infestation levels, especially in aphid colonies lacking their protective endosymbionts. *Bacillus* species and their target insect orders are depicted in Table 4. *Advantages and specificity:* An essential advantage of using *Bacillus*-based biocontrol for pests is their generally high specificity and safety. *Bacillus* insecticidal proteins typically target specific insect groups (often a narrow range of species within an order) due to the need for particular gut receptors, meaning they do minimal harm to non-target insects (like pollinators, predators, and other beneficials) and do not accumulate as toxins in the environment [99]. They are also biodegradable, breaking down into natural compounds, thus leaving no persistent residues on crops or in soil [100]. While pests can develop resistance to *Bt* toxins over time, the risk is mitigated by strategies like rotating strains or pyramiding multiple toxin genes (as done in transgenic Bt crops), and in general, the development of resistance to *Bacillus* biocontrols has been slower and less frequent compared to chemical insecticides [93].

**Table 4:** *Bacillus* species and their target insect orders

Insect order	Target pest(s)	*Bacillus* species	Mode of action	Examples of success	Reference (s)
Diptera	*Anopheles spp.*, *Culex spp.*, *Simulium spp.*	*Bacillus thuringiensis israelensis* (*Bti*)	Cry and Cyt toxins disrupt larval midgut cells	Control of mosquito larvae in stagnant water bodies	[14]
	*Culex quinquefasciatus*	*Bacillus sphaericus*	Binary toxin damages the midgut epithelium	Reduction of filariasis vector populations	[101]
Hymenoptera	Red imported fire ant (*Solenopsis invicta*)	*Bacillus subtilis*	Produces antifungal compounds	Suppression of invasive ant species	[102]
	Beneficial bees (disease management)	*Bacillus subtilis*	Antagonistic effects on pathogens	Protection against chalkbrood and foulbrood in bees	[102]
Hemiptera	Whitefly (*Bemisia tabaci*)	*Bacillus amyloliquefaciens*	Lipopeptides disrupt insect physiology	Control in greenhouse crops	[103]
	Brown marmorated stink bug (*Halyomorpha halys*)	*Bacillus cereus*	Insecticidal activity against feeding behavior	Reduction in crop damage in experimental studies	[104]
	Aphids (*Aphis spp.*)	*Bacillus thuringiensis*	Cry toxins target hemipteran larvae	Reduced aphid infestations in field crops	[105]
Lepidoptera	Diamondback moth (*Plutella xylostella*)	*Bacillus thuringiensis kurstaki* (*Btk*)	Cry toxins disrupt midgut cells in larvae	Effective control in cabbage and cruciferous crops	[106]
	Cabbage white butterfly (*Pieris rapae*)	*Bacillus thuringiensis*	Cry toxins induce gut paralysis in larvae	Reduced larval populations in field trials	[106]
	Fall armyworm (*Spodoptera frugiperda*)	*Bacillus thuringiensis aizawai*	Multiple Cry toxins target larval stages	Significant suppression of crop damage in maize	[34]
	Cotton bollworm (*Helicoverpa armigera*)	*Bacillus thuringiensis kurstaki* (*Btk*)	Cry toxins disrupt larval digestion	Effective control in cotton and tomato fields	[107]

#### Synergistic Effects with other Biocontrol Agents

*Bacillus* spp. often exhibits synergistic effects when combined with other biocontrol agents, enhancing disease management in crops [108]. These interactions can improve efficacy against a wide range of pathogens, including fungi, bacteria, and viruses [109]. For example, combining *Bacillus* with microorganisms such as *Trichoderma* spp. and *Pseudomonas* spp. can lead to enhanced biocontrol activity through mechanisms like the production of a broader spectrum of antimicrobial compounds and improved competition for nutrients and space in the rhizosphere [110, 111]. Additionally, the integration of *Bacillus* with mycorrhizal fungi has shown potential in boosting plant growth and resistance to diseases, as mycorrhizal associations enhance nutrient uptake, particularly phosphorus, while *Bacillus* contributes to disease suppression and growth promotion [112]. Furthermore, *Bacillus’s* ability to form biofilms enhances the stability and longevity of co-inoculated microbes, ensuring their sustained activity and reducing the need for repeated applications, ultimately lowering costs for farmers [113].

## *Bacillus* as Plant Growth Promoting Rhizobacteria (PGPR)

Beyond biocontrol, *Bacillus* species are renowned for their plant growth-promoting activities. As PGPR, they enhance crop growth and yield through a variety of direct and indirect mechanisms. These include improving nutrient availability (nitrogen fixation, phosphate and potassium solubilization, micronutrient chelation), synthesizing phytohormones that stimulate plant development, and mitigating abiotic stresses that would otherwise impair growth [114]. The net effect is often healthier, more vigorous plants with greater stress tolerance and higher productivity, even under suboptimal conditions. Incorporating *Bacillus* inoculants into crop production can thus reduce the need for chemical fertilizers and growth regulators, contributing to more sustainable agriculture [115].

### Nutrient Solubilization and Uptake Enhancement

One of the primary ways *Bacillus* promotes plant growth is by increasing the availability and uptake of essential nutrients in the soil [116]. Many *Bacillus* spp. are capable of biological nitrogen fixation while not as efficient as symbiotic rhizobia, free-living *Bacillus* in the rhizosphere can convert atmospheric nitrogen (N₂) into ammonia or related forms that plants can assimilate. This process supplements soil nitrogen, particularly in low-N environments, and can partly fulfill the crop’s nitrogen requirements [117]. *Bacillus* is often included in biofertilizer formulations to reduce dependence on synthetic N fertilizers. For example, inoculation of wheat and maize with nitrogen-fixing *Bacillus* strains has been shown to improve plant N content and grain yield, demonstrating its contribution to natural nitrogen inputs [118]. Phosphorus solubilization is another critical function. *Bacillus* spp. can liberate inorganic phosphate from insoluble compounds (such as rock phosphates or bound soil phosphates) by secreting organic acids (e.g., gluconic, lactic, or citric acid) and phosphatase enzymes that dissolve mineral phosphates. The lowering of pH in the microenvironment and enzymatic action releases phosphate ions that roots can absorb [119]. Studies indicate that *Bacillus* inoculation often leads to higher phosphorus uptake in plants and improved root and shoot growth, especially in P-deficient soils. In one case, *B. megaterium* application on soybean increased available soil P and boosted soybean P content and yield relative to uninoculated controls. Similarly, *Bacillus* enhances the cycling of other nutrients: certain strains can solubilize potassium from insoluble K-bearing minerals by producing acids that leach out K⁺, making it accessible to plants [116]. *Bacillus*-mediated K solubilization has been correlated with improved K nutrition and drought resistance in crops like maize [120]. Some *Bacillus* species also chelate micronutrients (iron, zinc) through siderophores and other ligands, thereby increasing the uptake of these micronutrients by plant roots. For instance, the siderophore bacillibactin not only suppresses pathogens but also supplies iron to the plant, which can alleviate iron chlorosis in calcareous soils [121].

Through these nutrient-oriented activities, *Bacillus* effectively acts as a biofertilizer. Crops treated with *Bacillus* inoculants often show enhanced nutrient content (N, P, K, Fe, Zn, etc.) in their tissues and corresponding improvements in growth metrics. Importantly, *Bacillus* can partially substitute chemical fertilizers: field experiments have demonstrated that combining *Bacillus* biofertilization with reduced levels of NPK fertilizers still maintains or improves yield. For example, a recent study in Chinese cabbage found that integrating *Bacillus* inoculation with a 25% reduction in chemical fertilizer resulted in equivalent or higher yields than complete fertilizer without *Bacillus*, due to better nutrient uptake and enriched beneficial rhizosphere microbes [122]. This highlights *Bacillus*’ role in sustainable intensification, improving nutrient use efficiency, and reducing fertilizer inputs.

### *Phytohormone Production and Stress Mitigation*

Another major PGPR trait of *Bacillus* spp. is the ability to produce and modulate phytohormones, which are chemical signals that regulate plant growth and development [123]. *Bacillus* strains have been documented to synthesize auxins (especially indole-3-acetic acid, IAA), cytokinins, gibberellins, and ethylene modulators, all of which can significantly influence plant physiology [124]. AA, a primary auxin produced by many *Bacillus* spp., stimulates root elongation and branching at low concentrations. *Bacillus*-derived IAA leads to larger root systems with more root hairs, thereby increasing the plant’s capacity for water and nutrient absorption [125]. Enhanced root development not only supports better nutrition but also improves anchorage and tolerance to drought. Cytokinins from *Bacillus* can promote cell division and shoot growth, often resulting in greater leaf area and delayed senescence (which prolongs photosynthetic activity). Gibberellins produced by *Bacillus* may contribute to stem elongation and seed germination processes, supporting early vigor of crops. The combined hormonal effects typically manifest as increased biomass, more flowers and fruits, and higher overall yield in various crops inoculated with *Bacillus* compared to controls [126]. In addition, *Bacillus* helps plants cope with abiotic stresses such as drought, salinity, and heavy metal toxicity. One way is through ethylene regulation. Under stress, plants often produce excess ethylene, a hormone that at high levels can inhibit root growth and cause premature senescence (the stress ethylene response) [127]. Some *Bacillus* strains carry the enzyme ACC deaminase, which breaks down ACC (1-aminocyclopropane-1-carboxylate), the immediate precursor of ethylene in plants. By lowering plant ACC levels, *Bacillus* thereby reduces stress-induced ethylene production, preventing the deleterious effects of ethylene on plant growth. This results in improved stress tolerance – for example, *Bacillus* with ACC deaminase activity can help maintain root growth in waterlogged or salt-affected conditions where ethylene would normally stunt roots [128]. In one study, engineering *B. amyloliquefaciens* to overexpress an ACC deaminase gene significantly improved the growth of waterlogged tomato plants, illustrating the principle [129].

Moreover, *Bacillus* induces plant production of osmoprotectants and antioxidants. Under drought or high salinity, *Bacillus*-inoculated plants commonly accumulate higher levels of proline, trehalose, and other osmoprotective solutes, as well as antioxidant enzymes (catalases, superoxide dismutases) compared to uninoculated plants [130]. This biochemical priming helps to maintain cellular functions and mitigate oxidative damage during stress. Field trials in arid regions have noted that crops (e.g., wheat, chickpea) treated with drought-tolerant *Bacillus* strains showed better moisture retention in tissues and sustained growth during dry spells relative to controls [131]. Likewise, under saline conditions, *Bacillus* can improve ion balance in plants, for instance by increasing K⁺ uptake over toxic Na⁺, partly through better root growth and perhaps hormone influences [132].

## Molecular and Omics Advances in *Bacillus* Research

Modern molecular biology and omics technologies have significantly advanced our understanding of *Bacillus* spp. and enabled the improvement of their biocontrol and PGPR traits. Researchers are unraveling the genetic basis of *Bacillus*’ beneficial activities and using genetic engineering to enhance these functions [115]. In parallel, genomics, transcriptomics, proteomics, and metabolomics (“omics” approaches) are providing system-level insights into how *Bacillus* interacts with plants and other microbes, guiding the development of more effective *Bacillus*-based applications. In this section, we discuss how genetic manipulation boosts *Bacillus* efficacy and how omics studies shed light on *Bacillus* mechanisms and facilitate strain selection and design.

### Genetic Basis of Biocontrol Traits and Engineering of *Bacillus* Strains

The ability of *Bacillus* to produce antimicrobial compounds, enzymes, and phytohormones is encoded by a diverse set of genes often organized in clusters or operons. For instance, genes responsible for non-ribosomal lipopeptide synthesis (such as *itu* for iturin, *fen* for fengycin, *srf* for surfactin) have been identified in various *Bacillus* genomes, and their regulation is linked to quorum sensing and stress-response pathways [133]. Advances in whole-genome sequencing have allowed researchers to pinpoint such biocontrol-related gene clusters. Comparative genomics across *Bacillus* isolates has further highlighted that certain strains carry unique genes (or gene variants) that may confer superior biocontrol or PGPR capabilities. This helps in selecting elite strains for specific agricultural applications (e.g., a strain with an extra chitinase gene might be perfect against fungal pathogens) [134]. Building on this genomic knowledge, scientists have begun genetic engineering of *Bacillus* to enhance desirable traits. Traditional mutagenesis and selection have been used to obtain *Bacillus* mutants with higher antibiotic production or better root colonization [135]. More recently, precise genetic modifications are possible thanks to CRISPR-Cas and other tools being adapted to *Bacillus*. For example, researchers have deleted specific regulatory genes (encoding repressors of secondary metabolism) in *B. subtilis*, which led to significantly increased production of fengycin and iturin lipopeptides. By removing negative regulators, the biosynthetic pathways are derepressed, yielding hyperproducer strains that show more potent pathogen inhibition in assays. On the flip side, genes that positively influence biocontrol traits can be overexpressed. Notable case is the introduction of hybrid cry genes into *B. thuringiensis* to broaden its insecticidal spectrum. By combining two toxin genes (*cry1Ac/cry2Ab*), engineered *Bt* strains can produce multiple Cry proteins and thereby kill a broader range of Lepidopteran pests, including those that might be resistant to a single toxin [136, 137].

Similarly, *B. amyloliquefaciens* has been genetically modified to overproduce siderophores, enhancing its iron-scavenging ability and thus its pathogen-suppressive effect in iron-limited soils. This also boosts the strain’s plant-colonization competitiveness and nutrient provision to the plant. Another genetic improvement is the insertion of the acdS gene (ACC deaminase) from other bacteria into *Bacillus*, which endowed the modified strains with the capacity to lower plant ethylene levels and protect plants under stress. Such a transgenic *Bacillus* demonstrated better promotion of root growth in flooded conditions compared to the wild type [129]. Quorum-sensing genes have also been targets altering the *comQXPA* quorum-sensing system in *B. subtilis* led to enhanced biofilm formation and root colonization, as this global regulator coordinates the expression of biofilm matrix genes and other factors. The engineered strain showed improved persistence in the rhizosphere and longer-lasting biocontrol activity [138]. In terms of nutrient mobilization, *Bacillus megaterium* was engineered to overexpress a glucose dehydrogenase gene (for improved phosphate solubilization via gluconic acid production), resulting in higher soluble phosphate and better plant P uptake in trials [139]. Other enhancements include boosting *Bacillus* production of volatile organic compounds like acetoin and 2,3-butanediol, which have roles in plant growth promotion and ISR triggering. This was achieved by modifying key metabolic enzymes in those pathways [140]. There are even experimental efforts to create synthetic consortium strains for instance, *Bacillus* strains engineered with *Trichoderma* antifungal genes or vice versa aiming to combine multiple biocontrol modes in one organism (though regulatory approval for such GMOs in open field use is a consideration) [141].

The upshot of these genetic engineering endeavors is a new generation of *Bacillus* bioinoculants with enhanced efficacy and reliability. Early greenhouse tests with some engineered strains have shown promise, such as significantly better protection against fungal root rot by a *Bacillus* overproducing antifungal lipopeptides, compared to its wild type. It should be noted that regulatory and public acceptance issues surround GMO microbes; thus, current practical use still relies mainly on natural or conventionally improved *Bacillus* strains. Nonetheless, the knowledge gained from genetic studies is invaluable for guiding non-GMO approaches as well. For example, knowing which genes are critical allows for marker-assisted selection of natural isolates or for formulating combinations that complement each other’s genetic capabilities [142].

### Omics Technologies Unraveling *Bacillus* Plant Environment Interactions

The application of omics technologies has provided deep insights into how *Bacillus* functions in complex environments, interacts with plants, and responds to stress or competition [143] These approaches enable researchers to capture snapshots of *Bacillus* at the DNA, RNA, protein, and metabolite levels, leading to a systems-level understanding that can inform more targeted use of *Bacillus* in agriculture [144].

#### Genomics

As mentioned, whole-genome sequencing of numerous *Bacillus* strains has identified key genetic determinants of biocontrol and PGPR activity. Comparative genomics can reveal why one strain is a better biocontrol agent than another by highlighting unique genes or gene variants [145]. For instance, comparative genome analysis of an effective biocontrol *B. velezensis* strain might show the presence of an extra NRPS (nonribosomal peptide synthetase) gene cluster for a novel antibiotic that fewer effective strains lack. Such insights allow screening for those genetic markers in strain discovery programs. Pangenomic studies across dozens of *Bacillus* isolates are delineating the core genome (shared by all, often housekeeping genes) versus the accessory genome (variable genes, often including secondary metabolite clusters) [146]. Interestingly, many biocontrol and PGPR genes fall in the accessory genome, explaining the functional diversity among strains. Genomics has also facilitated the design of strain-specific primers/probes to track *Bacillus* inoculants in the field and ensure they are establishing as intended [5].

#### Transcriptomics

By examining the messenger RNA profiles, transcriptomic studies reveal which genes *Bacillus* turns on or off under particular conditions (in the presence of a plant root vs. alone in soil, or during confrontation with a pathogen). This helps decipher *Bacillus*’ mode of action. Research conducted RNA-seq on *B. amyloliquefaciens* during interaction with a plant pathogen and found upregulation of specific gene clusters linked to antibiotic production and motility, indicating the bacterium was mounting a defensive/offensive response [147]. Another study on *B. thuringiensis* transcriptomes identified regulatory genes activated upon sensing the insect gut environment, shedding light on how *Bt* adapts to infect insect larvae effectively. Such knowledge can guide the development of *Bacillus* strains that express biocontrol genes at the right time – for instance, engineering a promoter to be induced by plant root exudates, ensuring the genes for antibiotics or ISR elicitors are expressed when *Bacillus* is near the host plant [148]. Transcriptomics also helps in understanding plant responses; some studies simultaneously profile plant gene expression when inoculated with *Bacillus*, revealing which plant defense pathways are triggered, thereby confirming the induction of systemic resistance and highlighting key marker genes for that process [149].

#### Proteomics

Proteomic analyses focus on the actual proteins produced by *Bacillus*, which are the workhorses executing biocontrol and growth promotion. By comparing the proteome (all proteins expressed) of *Bacillus* under different conditions, researchers can identify necessary enzymes and toxins [150]. For instance, proteomic comparison of *B. subtilis* under pathogen challenge versus control showed increased abundance of chitinase, β-glucanase, and surfactin synthetase proteins in the challenged condition, directly confirming these proteins’ role in antagonism [151]. Proteomics can also uncover post-translational modifications or secretion of proteins that transcriptomics might miss (because mRNA levels don’t always reflect protein levels due to regulatory mechanisms). An outcome of proteomics is the formulation of improved bioproducts e.g., if a protein critical for root colonization is identified, one might formulate the *Bacillus* product with additives that enhance the expression or stability of that protein [152]. Proteomics has also been used to analyze *Bacillus* formulations, verifying that they produce the expected set of antifungal proteins in storage or after application. Furthermore, by examining the secreted proteome (secretome), scientists have cataloged a variety of *Bacillus* enzymes and peptides released into the environment, which can inform on how *Bacillus* fights pathogens or aids plants (for example, detecting secreted ACC deaminase or phytase gives direct evidence of those functions in situ [153].

#### Metabolomics

Metabolomics provides a comprehensive profile of the small-molecule metabolites produced by *Bacillus*. This is particularly valuable for identifying novel antibiotics, siderophores, or phytohormones in *Bacillus* cultures. Through techniques like HPLC, mass spectrometry, and NMR, researchers have discovered previously unknown cyclic lipopeptides and polyketide compounds in *Bacillus* that have potent antifungal or antibacterial activity [154]. For example, metabolomic screening of a *B. cereus* strain revealed it produced a unique antifungal metabolite effective against certain soil-borne fungi. Additionally, metabolomics has been used to detect VOCs emitted by *Bacillus* in the rhizosphere; compounds like acetoin, 2,3-butanediol, and certain terpenes were identified, which are known to stimulate plant growth and ISR. Understanding the metabolite spectrum, scientists can sometimes optimize growth media or conditions to boost the production of a desired metabolite in fermentation (improving commercial formulation yields) [134, 140]. Metabolomics also assists in monitoring *Bacillus* activity in the field: soil samples from *Bacillus*-treated plots can be analyzed for the presence of specific lipopeptides, confirming that the bacteria are metabolically active and producing biocontrol agents in situ [155].

## *Bacillus* in Sustainable Agriculture: Ecological and Safety Implications

### Role of *Bacillus* in Sustainable Agriculture

*Bacillus* spp. are essential in sustainable agriculture since they improve crop yield and decrease the use of chemicals. hey control plant pathogens, thereby reducing the need for synthetic pesticides [85], which not only lowers environmental pollution but also protects beneficial soil microorganisms that influence soil quality [156]. In addition to pathogen suppression, *Bacillus* spp. play a key role in nutrient cycling and maintaining soil fertility. They dissolve phosphate and fix nitrogen, making nutrients more available to plants and increasing growth and yield [49]. This natural fertilization process significantly reduces dependence on chemical fertilizers [157]. Moreover, *Bacillus* modifies soil conditions and structure through the synthesis of extracellular polysaccharides and biofilms, which improve the thixotropic behavior of microaggregate soil. These enhancements support better root penetration and nutrient absorption, thereby improving plant health and resilience [158]. The presence of *Bacillus* in the rhizosphere promotes a balanced microbial population, contributing to the structural and functional integrity of the soil ecosystem. Furthermore, *Bacillus* spp. plays a crucial role in mitigating abiotic stresses such as drought and salinity challenges that are increasingly relevant due to climate change. *Bacillus* helps plants maintain cellular homeostasis under adverse conditions, improving crop resilience [159]. This stress mitigation capability is vital for maintaining crop productivity in challenging environments.

### Environmental Impact and Safety

Using *Bacillus* spp. in agriculture offers significant environmental benefits and safety advantages compared to conventional chemical methods. Bacilli used in biocontrol are eco-friendly, eliminating the possibility of polluting groundwater and soil, unlike synthetic pesticides and organic fertilizers [160]. Chemical input is lowered, thereby minimizing negative impacts on non-target organisms such as beneficial insects, earthworms, and soil microorganisms that play critical roles in maintaining ecological balance. Another merit of *Bacillus* spp. is their role in the biodegradation of organic matter, promoting soil health and fertility through substrate degradation, nutrient cycling, and facilitation of plant nutrition [161]. This transformation also helps control the buildup of organic waste, making the ecology of the agricultural sector more sustainable. From a safety point of view, *Bacillus* spp. is considered safe (GRAS) for use in agriculture and food production. Due to their non-pathogenic nature and beneficial properties, they have long been used in various applications, including probiotics for humans and animals. Their safety profile makes them suitable substitutes for chemical inputs, especially in organic farming, where chemical applications are prohibited. Moreover, *Bacillus* formulations are relatively easy to produce and apply, making them accessible to small-scale and resource-limited farmers. Their adaptability to different environmental conditions ensures effectiveness across diverse agricultural landscapes, supporting broader adoption of sustainable practices. *Bacillus* improves crop productivity and aligns with environmental conservation goals, promoting a holistic approach to agricultural sustainability[156].

## Conclusions

*Bacillus* spp. serves as a powerful microbial ally in advancing sustainable agriculture, offering multifunctional roles as biocontrol agents, plant growth promoters, and ecological stabilizers. Their ability to produce a diverse repertoire of bioactive compounds, including lipopeptides, antibiotics, enzymes, and phytohormones, enables them to suppress a broad spectrum of pathogens while simultaneously enhancing nutrient uptake, stress resilience, and soil fertility. These traits, combined with their capacity for biofilm formation, root colonization, and induced systemic resistance, position *Bacillus* spp. as key microbial resources in integrated crop management systems. Recent steps in omics technologies have unraveled the genetic and metabolic networks underpinning *Bacillus*-mediated plant–microbe-pathogen interactions, offering novel avenues for microbial strain improvement through genetic engineering. However, despite promising field results, challenges such as inconsistent performance across agroecological zones, limited shelf life of formulations, and regulatory hurdles persist. Future research should prioritize the development of robust bioformulations, exploration of synergistic microbial consortia, and precision agriculture tools to predict and optimize *Bacillus* efficacy. Ultimately, *Bacillus* spp. hold significant promises for reducing chemical inputs, promoting agroecological sustainability, and contributing to climate-resilient farming. Harnessing their full potential requires a multidisciplinary approach that integrates microbial ecology, biotechnology, and system-level modeling to achieve productive, resilient, and environmentally sound agriculture.

### Challenges and Future Prospects

While *Bacillus* spp. offers immense potential for sustainable crop productivity, several limitations still require attention. One significant challenge is their inconsistent biocontrol efficacy across various climates, soil types, and crop systems, necessitating tailored application strategies [160]. Formulation and delivery issues are the main hurdles, including short shelf life and reduced microbial viability. Innovations such as microencapsulation and protective carriers are being explored to improve product stability and field performance. Additionally, complex and region-specific regulatory frameworks can delay commercialisation. Harmonisation of these standards would facilitate broader use of *Bacillus*-based products [162]. Despite these constraints, the future prospects for *Bacillus* spp. are strong. Advances in omics technologies and genetic engineering are enabling the development of strains with enhanced biocontrol traits and improved environmental resilience. Furthermore, the rising demand for eco-friendly agricultural practices supports the adoption of *Bacillus* in integrated pest management systems [163]. Key areas for future focus include enhancing formulation technologies, investigating synergistic interactions with other beneficial microbes, and tailoring *Bacillus* applications to specific agroecosystems. As sustainability pressures increase, *Bacillus* spp. is anticipated to play a central role in reducing chemical inputs and improving crop yields in the new agricultural era [164]. Although *Bacillus* spp. have long been explored as plant biocontrol agents, the integration of next-generation technologies and precision agriculture is now unlocking new dimensions. Recent insights reveal that *Bacillus*-mediated responses are strongly genotype-specific, suggesting the need for customized microbial formulations based on crop varieties and regional variables [165]. Moreover, the convergence of multi omics approaches linking transcriptomics, metabolomics, and metagenomics is providing a systems-level understanding of *Bacillus*-host–pathogen interactions and offering a roadmap for predictive biocontrol models [15]. Several key unexplored future avenues include.

#### Bioformulation Stability Enhancements

Integration of nanoencapsulation techniques, such as chitosan- or alginate-based nanocarriers, to extend shelf life and enable targeted delivery of *Bacillus* spores and metabolites [166].

#### Synthetic Microbial Communities (syncoms)

Designing synergistic consortia of *Bacillus* with compatible PGPRs like *Trichoderma* and *Pseudomonas* to develop multifunctional biocontrol cocktails suitable for climate-resilient farming systems [115].

#### AI-driven Decision Support Systems

Application of machine learning to predict the efficacy of *Bacillus*-based products across different agroclimatic zones, leveraging soil microbiome data and weather forecasts [167].

#### Carbon Sequestration and Climate Mitigation

Emerging studies suggest that *Bacillus* spp. may indirectly support carbon sequestration by stimulating root exudates that help stabilize organic carbon in rhizosphere soils, offering potential climate co-benefits [168].

## Data Availability

No original data were generated during this research and are not applicable.
